# Pleural Effusion as a Potential Complication of Foreign Body Reaction to Silicone Breast Implants: A Case Study

**DOI:** 10.7759/cureus.38734

**Published:** 2023-05-08

**Authors:** Yazan Abdeen, Wendolin J Ortiz, Juan D Cala-Garcia, Mario Cervantes

**Affiliations:** 1 Pulmonary and Critical Care Medicine, HCA (Hospital Corporation of America) Houston Healthcare Pearland, Houston, USA; 2 Pulmonary and Critical Care Medicine, Pulmonary and Sleep Physicians of Houston, Webster, USA; 3 General Surgery, Universidad Autónoma de Baja California, Mexicali, MEX; 4 Pathology, HCA (Hospital Corporation of America) Houston Healthcare Pearland, Houston, USA; 5 Pulmonary, Baylor College of Medicine, Houston, USA; 6 Pathology, HCA (Hospital Corporation of America) Houston Healthcare West, Houston, USA

**Keywords:** steroids, exudative pleural effusion, foreign body reaction, breast implant complications, left sided pleural effusion

## Abstract

Breast augmentation surgery, like any other surgery, has potential complications, including the less common complication of pleural effusion. We present a unique case of a 44-year-old female who developed pleuritic chest pain and shortness of breath 10 days after her breast augmentation surgery, with no prior history of cardiac or autoimmune conditions. The temporal relationship between the surgery and the onset of symptoms suggested a possible direct link to the implants. Imaging showed a small- to moderate-sized left pleural effusion, and pleural fluid analysis revealed findings suggestive of a foreign body reaction (FBR), including evidence of mesothelial and inflammatory cells with a lymphocyte percentage of 44% and monocytes of 30%. The patient received intravenous steroids at a dose of 40 mg every eight hours for three days while hospitalized, followed by a tapered oral dose of steroids upon discharge, for over three weeks. Follow-up imaging studies showed complete resolution of the pleural effusion. The diagnosis of pleural effusion resulting from FBR to silicone gel-filled breast implants involves a clinical history, cytopathological examination, and the exclusion of other potential causes. This case highlights the importance of considering FBR as a potential cause of pleural effusion post-breast augmentation surgery.

## Introduction

Breast augmentation surgery is a common procedure, with nearly 300,000 surgeries performed per year in the United States [1]. It is the second most prevalent aesthetic surgery in women [2]. Breast augmentation surgery, like any invasive procedure, is associated with complications, the most common of which include bleeding such as the development of hematomas, infection, development of seroma, sensory alterations in the nipple or breast, implant rupture/rotation, capsular contracture, and galactorrhea [3]. Less common events include silicone-induced granulomas, mammary fibromatosis, or the development of tumors such as desmoid tumors or breast implant-associated anaplastic lymphoma [4]. Rare cases of benign pleural effusion have been reported following breast augmentation surgery with silicone breast implants.

## Case presentation

**Figure 1:**
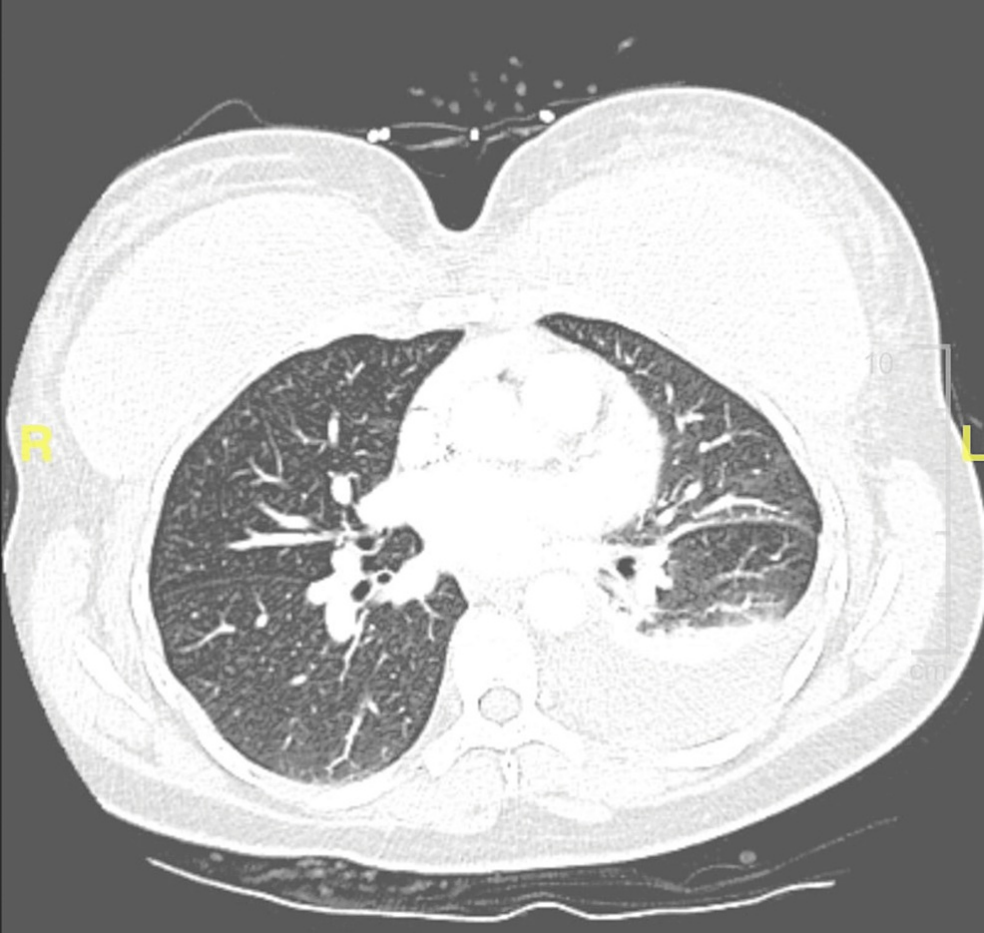
Computed tomography of the chest showing left pleural effusion

A 44-year-old female patient with a known medical history of hypertension presented to the emergency department with progressive pleuritic left-sided chest pain for 10 days. The pain was located at the anterolateral chest and extended to the back, associated with shortness of breath. The patient denied cough or fever at the time of presentation. The patient had undergone uncomplicated bilateral silicone breast implant placement two weeks prior. Three days after the procedure, the patient started having minimal left-sided chest discomfort that progressed to the level of unbearable pain, 10/10 in severity. The patient had no history of cardiac or autoimmune conditions. Physical examination on presentation showed normal vital signs, no swelling or redness of breasts, and a cleaned, healing surgical wound. Lung examination revealed reduced breathing sounds with dullness to percussion at the left base. Chest X-ray and computed tomography of the chest with contrast showed a small to moderate-sized left pleural effusion (Figure 1) and partial consolidation of the left lower lobe, which suggested atelectasis or pneumonia. No evidence of implant leak was observed.

**Figure 2:**
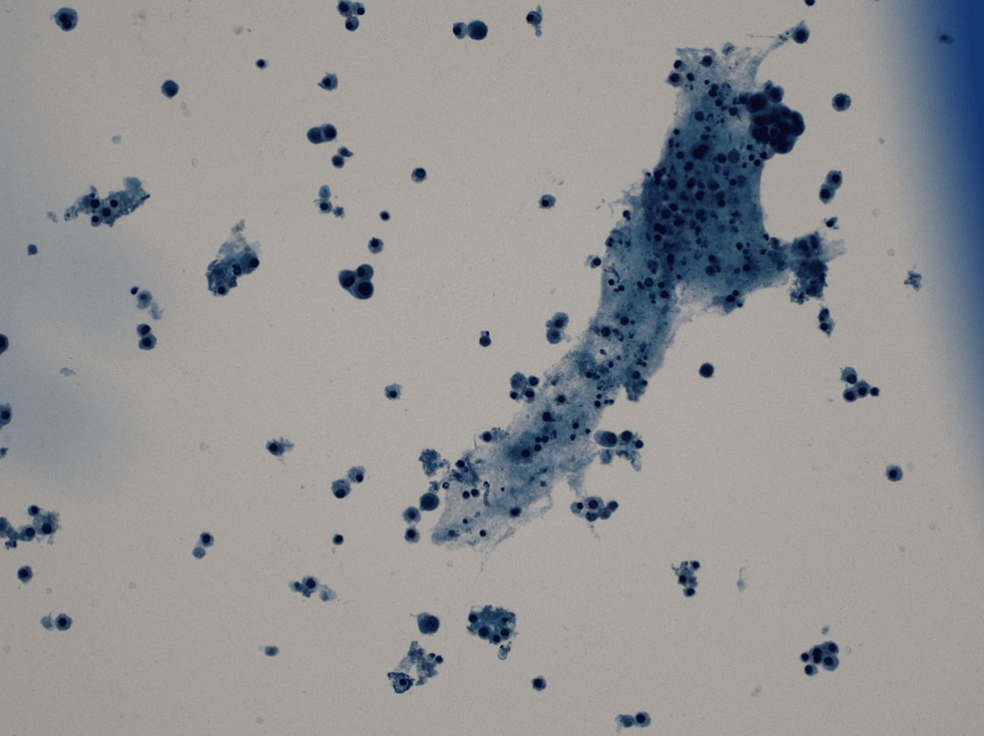
Cytological examination of pleural fluid

**Figure 3:**
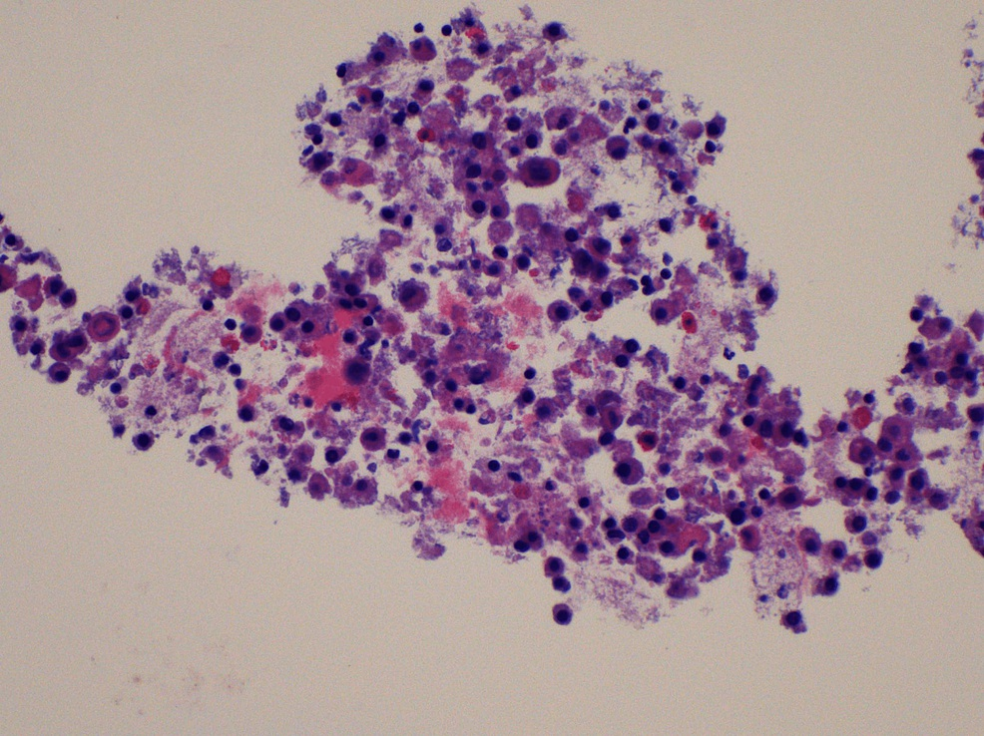
High-power view (60x magnification) of mesothelial and inflammatory cells in pleural fluid stained with hematoxylin and eosin

Complete blood count showed a normal WBC count of 6000 cells/mm³ (reference range: 3.8-9.8 k/mm³) and an erythrocyte sedimentation rate of 28 mm/hr (reference range: 0-20 mm/hr), and the workup for pneumonia, including sputum cultures and urine antigens for *Streptococcus pneumonia* and *Legionella pneumophila*, was negative. Left-sided thoracentesis was performed, yielding 350 mL of serosanguineous exudative fluid with a total protein level of 4.6 g/dL, lactate dehydrogenase level of 250 U/L, and normal glucose, amylase, and triglyceride levels. The cell count of the pleural fluid showed a WBC count of 2298 cells/mm³ (reference range: 0-10000 #/mm³) with a lymphocyte percentage of 44% (reference range: 50-100%) and monocytes of 30% (reference range: 0-10%). Pleural fluid cytology showed evidence of mesothelial and inflammatory cells, including numerous macrophages. No malignant cells were found (Figures 2, 3).

There was a concern for a silicone/foreign body reaction (FBR) to the implants causing a hyperinflammatory immune response without leaking, which could be reflected by reactive inflammatory pleural effusion fully responsive to steroids. The patient was started on intravenous steroids (40 mg every eight hours) for three days as an inpatient, followed by an oral taper of steroids over a three-week period (40 mg for five days, 30 mg for five days, 20 mg for 10 days, 10 mg for 10 days, then 5 mg for three days before stopping).

At the one-month follow-up, the patient reported complete resolution of symptoms, and follow-up imaging studies performed two months after treatment showed complete resolution of the pleural effusion.

## Discussion

We report the case of a patient with left-sided pleuritic chest pain with pleural effusion following a silicone breast implant surgery, despite no evidence of implant leak. Notably, the patient had no prior history of cardiac or autoimmune conditions, further supporting the likelihood of the effusion being directly linked to the breast implants.

Pleural effusion post-breast augmentation surgery was first described by Stevens et al. in 1987 [5]. Pleural effusion is one of the rarest complications for this type of surgery [5,6], and when found, underlying conditions such as anaplastic large cell lymphoma [7,8], implant rupture [5], or reactivation of latent infections [9] might explain the development of this complication. Shaik et al. described a case without any underlying condition, and after a pathologic evaluation that included an examination of the pleural fluid, they associated the pleural effusion with an FBR due to the presence of silicone particles and mononuclear cells [6].

After breast implant surgery, an FBR can occur as a result of immune system activation, particularly involving CD4+ cells, dendritic cells, and macrophages. These immune cells communicate closely with fibroblasts, which can result in increased production of collagenous and non-collagenous extracellular matrix proteins [10]. Capsular contracture is the most common manifestation of an FBR and is characterized by painful and firm breasts [11]. However, there is no known association between capsular contracture and pleural effusions.

Breast implant surgery can trigger an activation of the immune system that may result in complications other than capsular contractures. Chronic inflammation triggered by the presence of silicone in breast implants has been associated with immune deficiencies, autoimmune diseases, and neoplasias in patients who underwent this surgery. Autoinflammatory/autoimmunity syndrome induced by adjuvants (ASIA) is a systemic syndrome where many diverse symptoms such as fatigue, mind fog, hair loss, changes in weight, dry eyes, dry mouth, and depression develop after breast implant surgery [12,13].

It is reasonable to suspect FBR to silicone breast implants as the cause of our patient's pleural effusion based on its temporal relationship, cytology results, and response to steroids. Despite the absence of silicone in the pleural fluid and no evidence of implant rupture or leakage, FBR is a known complication of breast implants and can cause pleural effusion. The diagnosis of pleural effusion resulting from an FBR to silicone gel-filled breast implants involves a clinical history, cytopathological examination, and the exclusion of other potential causes. FBR is an inflammatory reaction caused by implanted materials, including breast implants, where tissue and inflammatory cells produce cytokines, chemokines, and matrix metalloproteinases. In cases of pleural effusion, cytology typically reveals mesothelial cells, macrophages, and lymphocytes, indicating FBR as the cause of the effusion [14].

Corticosteroids can be an effective treatment option for pleural effusions resulting from inflammation in certain conditions, such as autoimmune diseases and post-cardiac injury syndrome. The anti-inflammatory properties of corticosteroids are believed to reduce pleural fluid production and accelerate its absorption rate, leading to faster resolution of the effusion [15]. Studies have reported successful clearance of pleural effusions in seven days to six weeks with prednisone doses of 20 to 30 mg/day. Although no controlled studies have evaluated the efficacy of corticosteroids in treating rheumatoid pleural effusions, they may help prevent progressive pleural fibrosis. Spontaneous resolution of sarcoid pleural effusions can take one to three months, but steroid therapy has been reported to result in resolution as early as two weeks [16].

In our case, the patient's effusion completely resolved with steroid treatment, suggesting this could be a viable therapeutic approach for similar cases. However, long-term monitoring is recommended given the potential for autoimmune diseases and neoplasias associated with breast implants.

## Conclusions

Silicone breast implant-associated pleural effusion is a rare but important complication that should be considered in patients with pleural effusion of unknown etiology following breast surgery. This case highlights the need for careful evaluation and exclusion of other potential causes of pleural effusion, as well as the importance of recognizing the potential for FBR to silicone implants. Although the prevalence of this complication is relatively low in the literature, accumulating more data on the diagnosis and treatment of this condition is crucial to improving patient outcomes. Additionally, systemic corticosteroids may be effective in treating silicone breast implant-associated pleural effusion, and further studies are needed to evaluate the optimal management of this condition.
